# A bibliometric analysis of macrophage-associated immune regulation in atherosclerosis: advances in the mechanisms of pathogenesis

**DOI:** 10.3389/fimmu.2025.1559360

**Published:** 2025-06-12

**Authors:** Meiling Jiang, Tianci Han, Xianhui Li, Guofu Zhu

**Affiliations:** Cardiology Department, The Second Affiliated Hospital of Kunming Medical University, Kunming, Yunnan, China

**Keywords:** bibliometric analysis, atherosclerosis, macrophages, mechanisms, immune regulation

## Abstract

**Background:**

The occurrence and development of atherosclerosis (AS) is closely related to immune regulation. Macrophages serve as the primary immune cells involved in AS. However, the mechanisms underlying macrophage-mediated immune regulation in AS remain inadequately understood, necessitating the development of novel immunotherapeutic strategies. This article aims to review the current status and emerging trends in macrophage immune regulation related to AS on a global scale.

**Methods:**

We utilized the Web of Science Core Collection database to identify articles pertaining to macrophage immune regulation in AS published between 2000 and 2024. Bibliometric methods were used to analyze authors, institutions, countries, journals and references through CiteSpace and VOSviewer. A total of 1469 articles were included in this study.

**Results:**

The United States has published the highest number of articles in this field, followed closely by China. Maastricht University stands out as a leading institution specializing in macrophage immune regulation related to AS. Esther Lutgens from Germany has made significant contributions to this area of research. The authors identified “Arteriosclerosis, Thrombosis, and Vascular Biology” as the most influential journal within this domain. Through cluster analysis, the keywords were categorized into four primary groups: (1) autoantibodies, (2) activation, (3) immune activation, and (4) nuclear receptors.

**Conclusion:**

This study systematically summarizes the findings of macrophage immune regulation research in AS from 2000 to 2024, while also describing and predicting global research hotspots and trends. The investigation into the molecular mechanisms underlying macrophage immune regulation in AS is poised to become a prominent topic in future studies.

## 1 Introduction

Atherosclerotic cardiovascular disease (ASCVD) is a predominant cause of mortality and morbidity globally (1). Atherosclerosis(AS) is characterized by the development of plaques composed of lipids, connective tissue, and immune cells within the intima of large and medium-sized arteries (2). The significant reduction in cardiovascular mortality over the past three decades has primarily been achieved through programs and therapies aimed at lowering low-density lipoprotein cholesterol, as well as addressing other traditional risk factors for CVD, including hypertension, smoking, diabetes, and obesity. However, the overall benefits of targeting these risk factors have plateaued, and the global burden of CVD continues to persist (3). Recent evidence indicates that autoimmune mechanisms may play a significant role in the pathophysiology of AS (4).In recent years, the critical role of immune components in both the initiation and chronic progression of AS has gained prominence in clinical research.

The immune cell types involved in AS include T cells, B cells, natural killer (NK) cells, NKT cells, macrophages, monocytes, dendritic cells (DCs), neutrophils, and mast cells (5). Among these cell types, macrophages play a pivotal role in sustaining the inflammatory response, facilitating plaque formation, and promoting thrombosis. An increasing body of research has indicated that immune responses mediate alterations in macrophage function at all stages of AS (6). Consequently, macrophages represent key target immune cells for therapeutic interventions aimed at this condition (7). The relationship between the immune system and AS is intricate; typically, modulation of the immune response leads to attenuation of the disease process (8). Tolerogenic immunization shows promise as an effective strategy to induce protective adaptive immunity in patients suffering from AS- offering durability and relative cost-effectiveness (9). In the framework of predictive, preventive, and personalized medicine (PPPM), the advancement of novel and robust immunotherapies will significantly enhance management strategies for AS (10).

Bibliometric research is a quantifiable technique used to analyze academic literature, providing a comprehensive overview of specific topics. It highlights active authors, organizations, publications, influential contributions, and international collaborations (11).Song (12) et al. employed bibliometrics to analyze the knowledge base on macrophage polarization and trends in AS research. This study employed bibliometric methods to analyze articles pertaining to the immune regulation of macrophages in AS from 2000 to 2024. It summarizes the current research trends regarding the immune regulatory mechanisms associated with macrophages in AS and outlines key information in the field and assesses research topics, emerging hotspots, and their evolution to identify future trends.

## 2 Materials and methods

### 2.1 Database and search strategy

**Figure 1:**
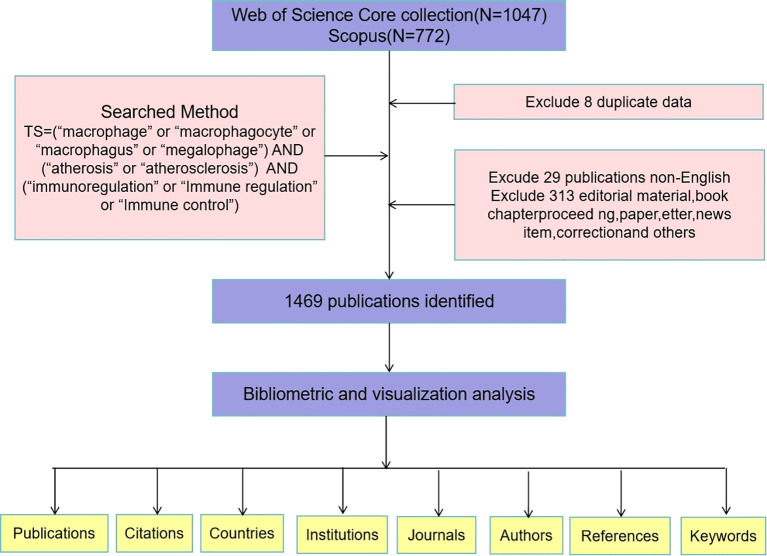
Detailed flowchart steps of the search strategy in screening publications.

Several biomedical databases are available for scientometric research, including Web of Science (WOS), Scopus, PubMed, MEDLINE, and Embase. While integrating these databases can create a more comprehensive dataset, it is crucial to recognize the significant amount of duplicate data present across them. Combining data from multiple sources may introduce confounding variables that could compromise data quality and affect experimental conclusions (13). All references in this paper are sourced from the Web of Science (WOS) and Scopus databases, which are recognized as the two most widely used resources for bibliometric analysis (14, 15). The selection of WOS and Scopus as the primary data source is justified for several reasons: (1) WOS and Scopus offer more complete data coverage and a broader scope that is highly suitable for scientific research; (2)WOS and Scopus provide detailed search queries that facilitate more accurate access to required information; (3) WOS and Scopusenjoy widespread recognition and usage globally and has been acknowledged as a critical foundation for scientific development planning and academic ranking within both domestic and international scholarly communities (16). **Figure 1** illustrates the specific data retrieval techniques and inclusion processes employed in this study. The search strategy is depicted in **Figure 1**. The articles included were published between 1 January 2000 and 30 November 2024.

### 2.2 Data reliability

In the data extraction process, rigorous verification procedures were implemented to ensure the accuracy and reliability of the extracted data. (1) Two independent reviewers conducted separate data extractions, gathering information such as journal source, title, author, research institution, abstract, and keywords from electronic databases while adhering to predefined standards to minimize bias. (2) After extraction, both reviewers cross-verified the data independently to identify and correct potential entry errors or inconsistencies. In cases of discrepancies or uncertainties about specific items, they referred back to the original literature for confirmation. (3) To ensure transparency and traceability throughout the process, each step was meticulously documented. This facilitates timely issue resolution during research and allows other researchers to effectively review and replicate our validation processes.

### 2.3 Data analysis

The following information was extracted from each article identified in the electronic search: journal source, title, author, research institution, abstract, and keywords. The screened literature was exported from the online database in a plain text format. Using Journal Citation Reports (JCR), the impact factor was calculated for 2023. Excel (2024) software was utilized to analyze the number of articles published per year and to create corresponding charts. VOSviewer version 1.6.20 software was employed for bibliometric analysis (17). CiteSpace version 6.3.R1 software facilitated an examination of the cooperative network among countries, authors, and constructs through collinearity and centrality analyses (18). The parameters were configured as follows: the time interval spanned from 2000 to 2024 with annual intervals; authors, institutions, and keywords were designated as node types. The path selection method used was “pathfinger,” while all other values remained at their default settings. Cluster analysis was conducted using the LLR algorithm within CiteSpace to group keywords into clusters that form a keyword map—this visualization illustrates both the number of nodes in each cluster and their respective years of occurrence. Keyword occurrence analysis provides insights into trends within this field over time. From 2000 to 2024, data were segmented annually to assess keyword emergence throughout these two decades.

## 3 Results

### 3.1 Analysis of the number of publications and citations

**Figure 2:**
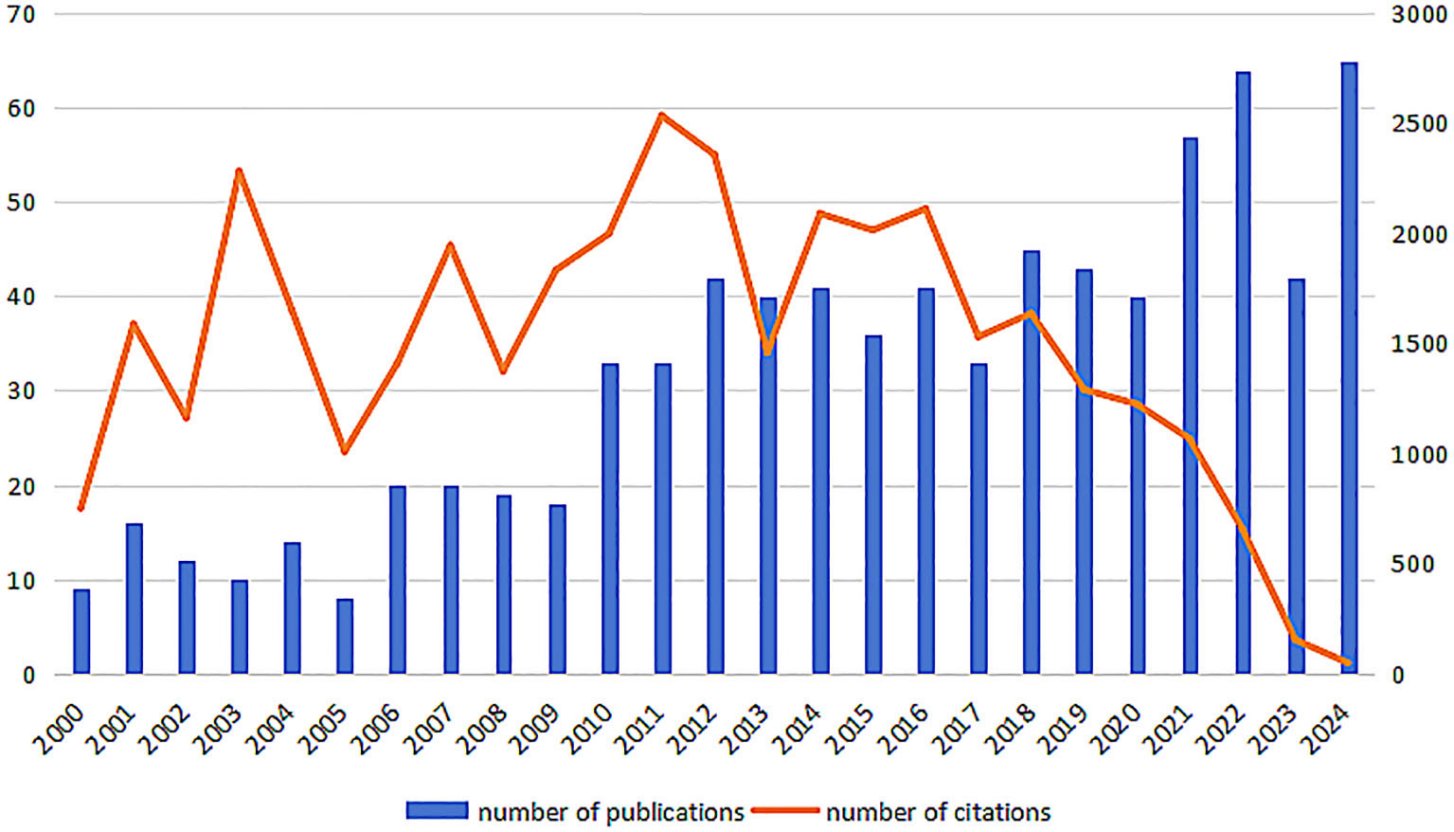
Annual number of publications and annual citations from 2000 to 2024.

According to a comprehensive manual review, there were 1469 papers published on the topic of AS and macrophage immune regulation from 2000 to 2024. The annual publication count and citation frequency are illustrated in **Figure 2**. It is evident that since the year 2000, the number of publications in this field has generally exhibited a fluctuating upward trend. With advancements in gene editing, single-cell sequencing, and related technologies, along with increased investment in research and closer international collaboration, there has been a significant boost in the generation of research outcomes. As a result, the number of published papers has shown an upward trend.

### 3.2 Analysis of contributions of prolific authors and co-cited authors

**Figure 3:**
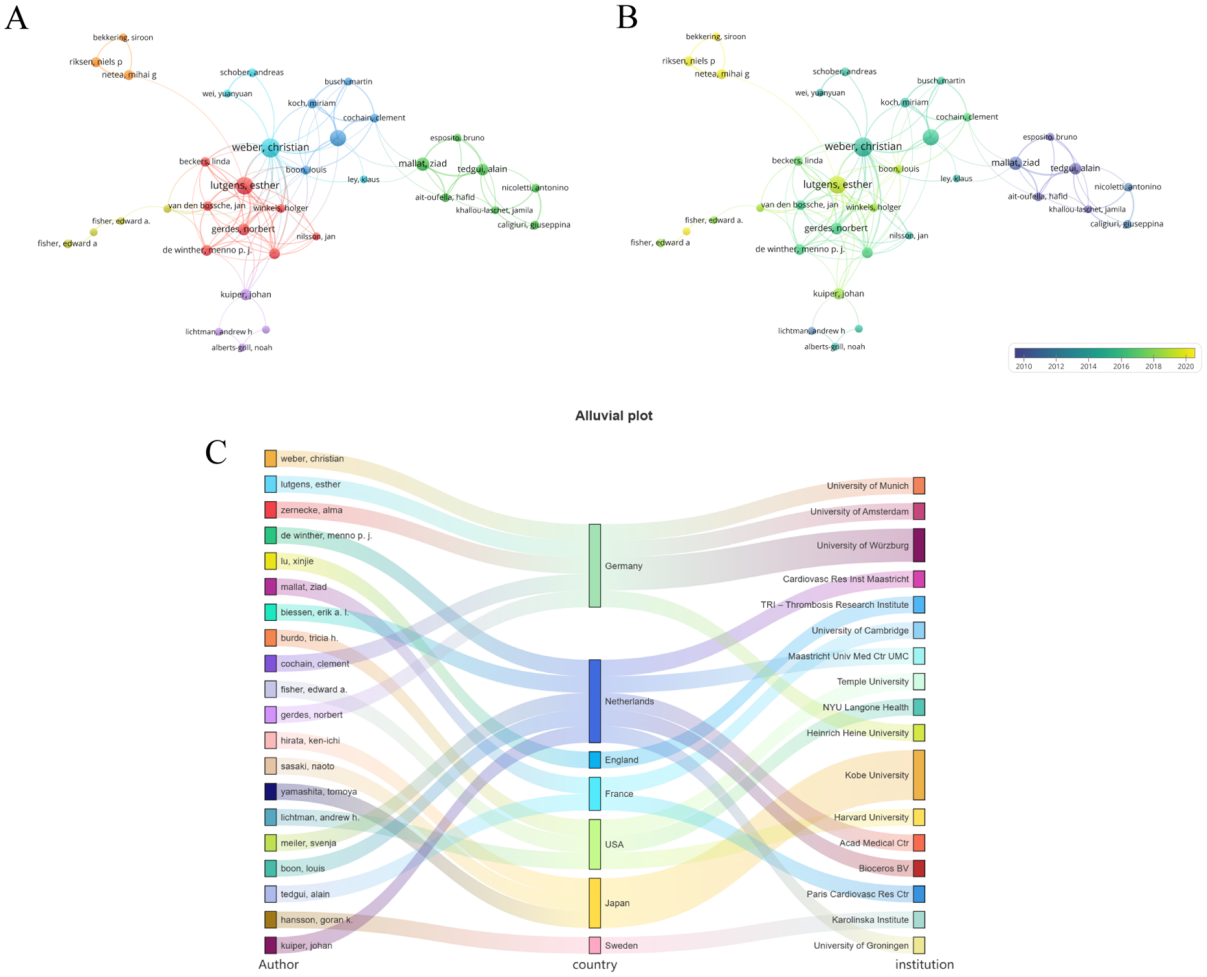
Co-authorship analysis of the excellent authors. **(A)** A network visualization map. **(B)** A overlay visualization map. **(C)** Countries and institutions of these distinguished authors. From: VOSviewer.

As illustrated in **Table 1**, the top 10 authors with the most publications in this field mainly come from four countries: Germany, the UK, the Netherlands, and Japan. Most of the leading co-cited authors hail from the United States, while others are from Sweden, Germany, France, and England. **Figure 3** presents a visualization of the collaborative network comprising authors who have published more than five papers; this network is characterized by eight distinct clusters (**Figure 3A**). Notably, Weber, Christian is the leading scholar in this field, with 28 published articles. The temporal chart clearly indicates that Esther Lutgens and Christian Weber from Germany have formed two new research groups (**Figure 3B**). In **Figure 3C**, we provide an overview of the top 20 authors along with their respective countries and affiliated institutions. This section elucidates the relationships between various constructs within this research landscape.

**Table 1:** Top 10 prolific authors and co-cited authors.

Rank	Author	Country	Documents	Citation	Average article citation	H-index	Co-cited author	Country	Total citation
1	weber, christian	Germany	28	2507	2014	19	hansson, gk	Sweden	421
2	lutgens, esther	Germany	22	933	2019	13	libby, p	USA	406
3	zernecke, alma	Germany	22	1263	2014	15	moore, kj	USA	224
4	mallat, ziad	England	16	1701	2013	14	ross, r	USA	199
5	hirata, ken-ichi	Japan	14	1074	2015	10	tabas, i	USA	196
6	yamashita, tomoya	Japan	14	1074	2015	9	ridker, pm	USA	186
7	sasaki, naoto	Japan	13	1013	2014	9	mallat, z	England	178
8	tedgui, alain	France	13	1500	2010	7	ait-oufella, h	France	146
9	gerdes, norbert	Germany	12	743	2018	10	swirski, fk	USA	138
10	kuiper, johan	Netherlands	12	889	2018	10	weber, c	Germany	132

### 3.3 Analysis of the contributions of journals

**Figure 4:**
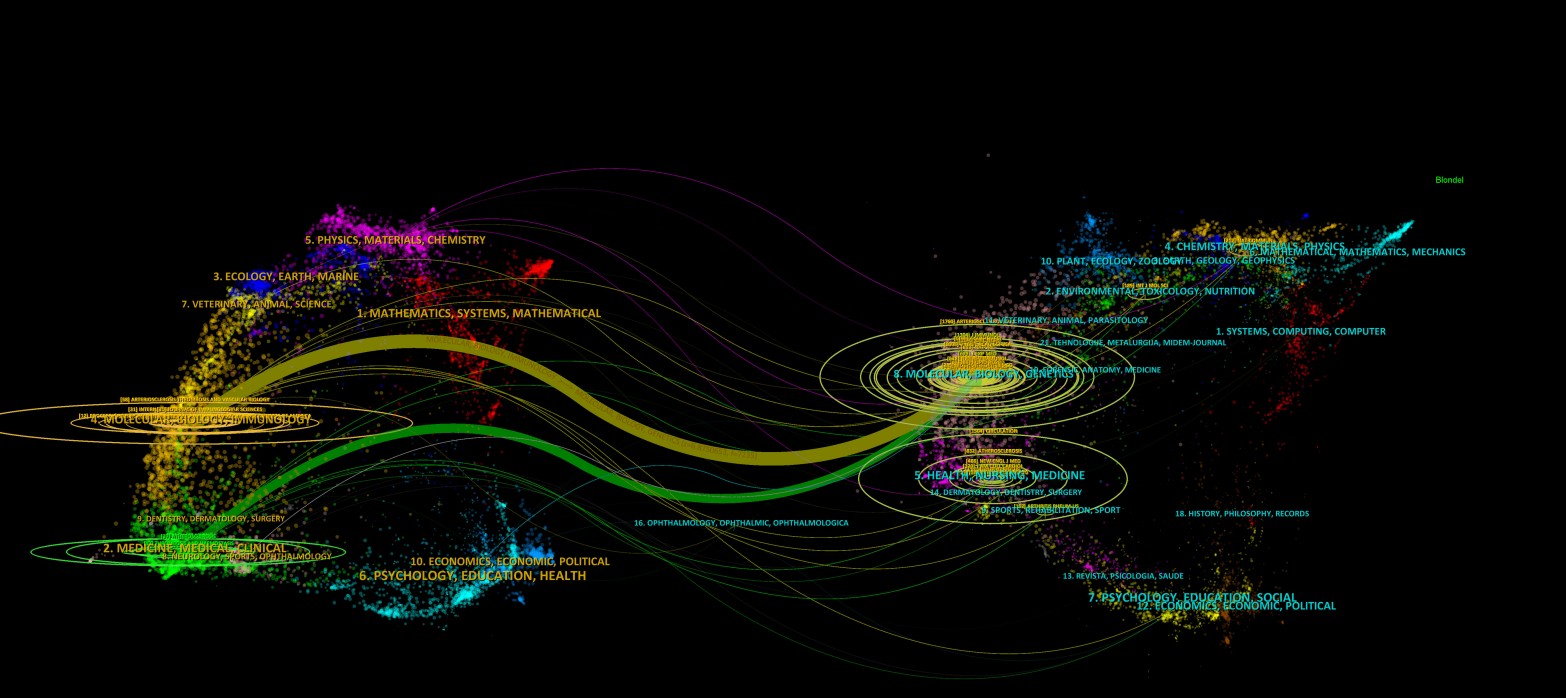
Double graph overlay of related journals.

**Figure 5:**
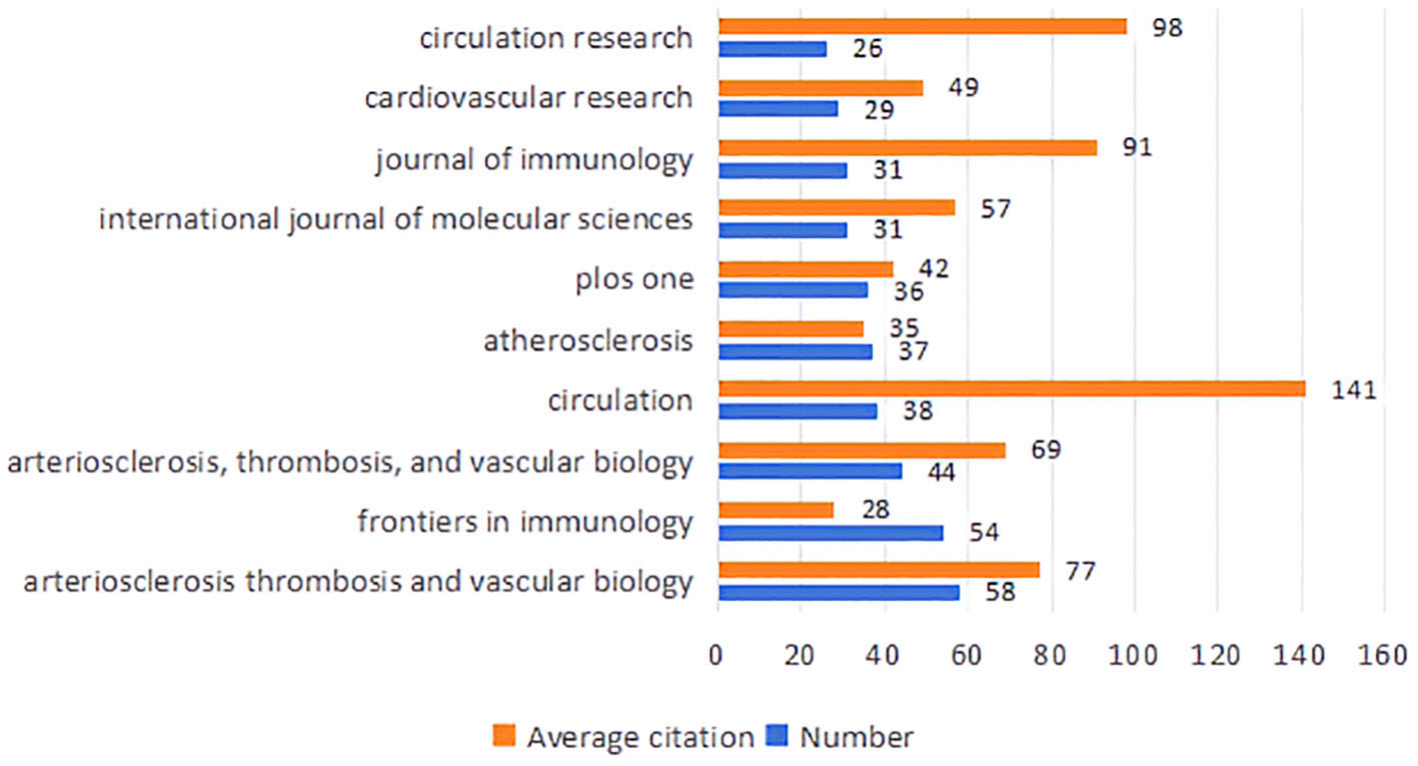
Average citations and total publications for the top 10 journals.

To enhance the clarity of citation distribution and the journals being cited, we employed a visualization technique involving the superimposition of double graphs representing different journals. As illustrated in **Figure 4**, the left section displays the distribution of cited journals, while the right section depicts the distribution of citing journals. The varying line tones within the figure correspond to distinct subject areas. Specifically, the cited journals predominantly span fields such as physics, ecology, mathematics, molecular biology, and medicine. Conversely, the citing journals primarily encompass disciplines including chemistry, botany, environmental science, molecular studies, health sciences, and sports. This dual graph representation facilitates a more intuitive understanding of citation relationships and knowledge flow across various disciplines. In total, 489 journals have published articles or reviews pertinent to this field. **Figure 5** presents an overview of average citations alongside total publications for the top ten journals ranked by publication volume. Notably among these top ten journals is a majority that falls within Q1 and Q2 subregions according to Journal Citation Reports (JCR).

### 3.4 Analysis of the contributions by institutions

**Figure 6:**
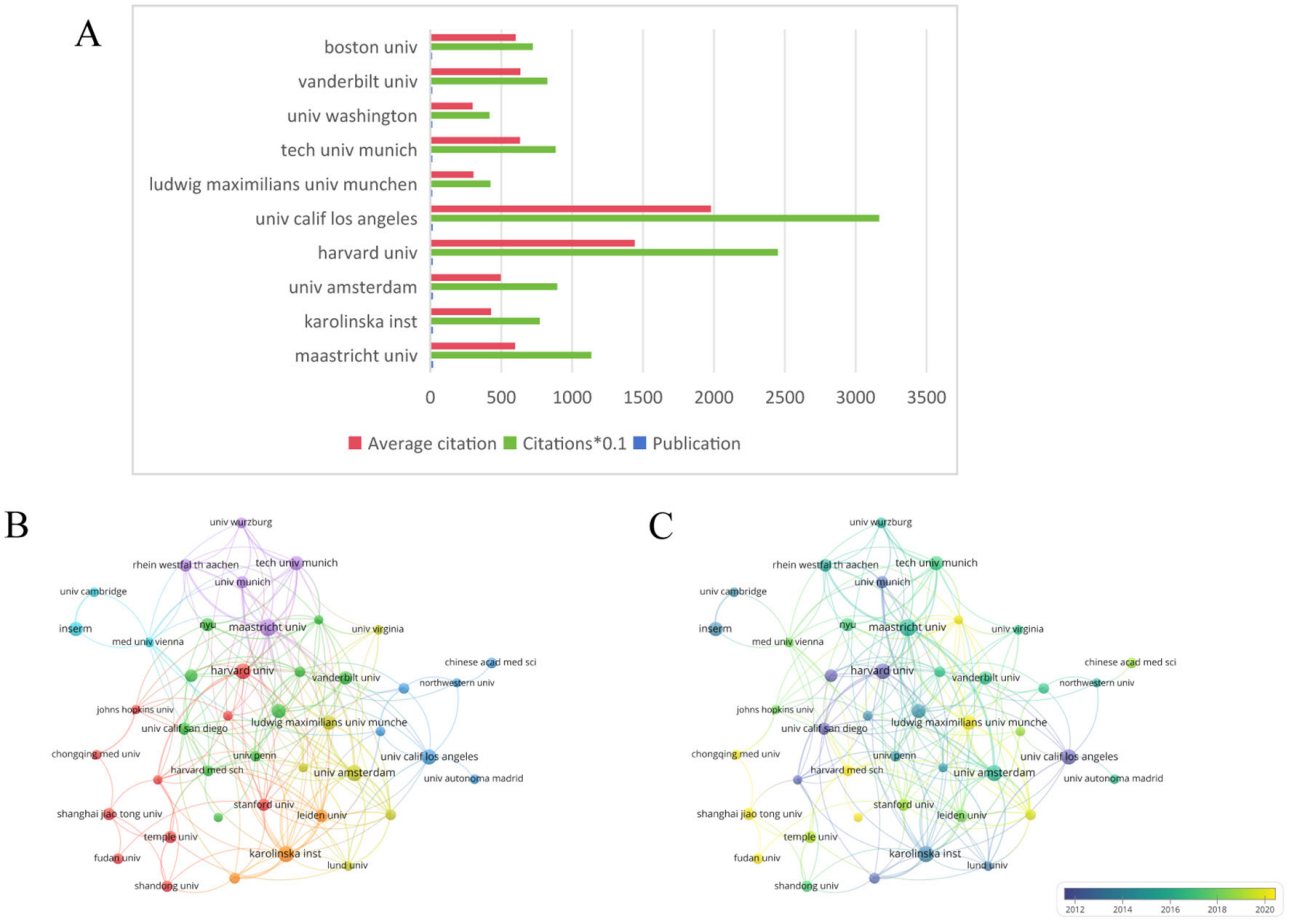
**(A)** The total number of publications, the average number of citations, visualization map. **(B)** A network visualization map. **(C)** A overlay visualization map. From: VOSviewer.

**Figure 6A** illustrates the top ten collaborating institutions based on research publications in this field. The VOSviewer software was employed to generate the institutional collaboration graph, as depicted in **Figure 6B**, where the minimum threshold for published papers required for institutional cooperation was set at five. The network graph of institutional collaboration comprises 116 institutions organized into 11 clusters. Furthermore, the temporal analysis of the institutional cooperation network presented in **Figure 6C** indicates that, in recent years, Chinese scientific research institutions have been increasingly influential in advancing research within this domain.

### 3.5 Analysis of the contributions of countries

**Figure 7:**
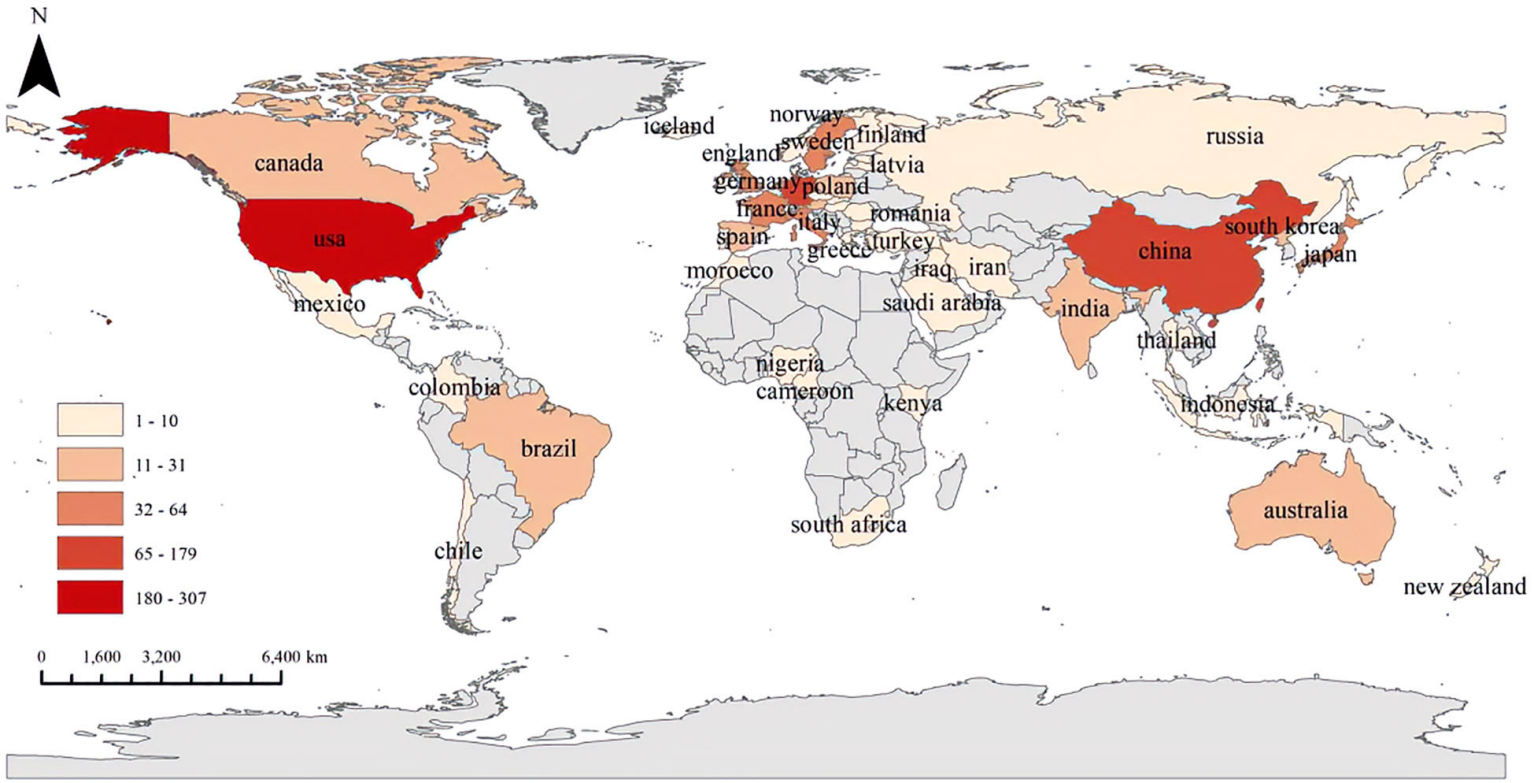
Geographical distribution of global publications of research in this field.

**Figure 8:**
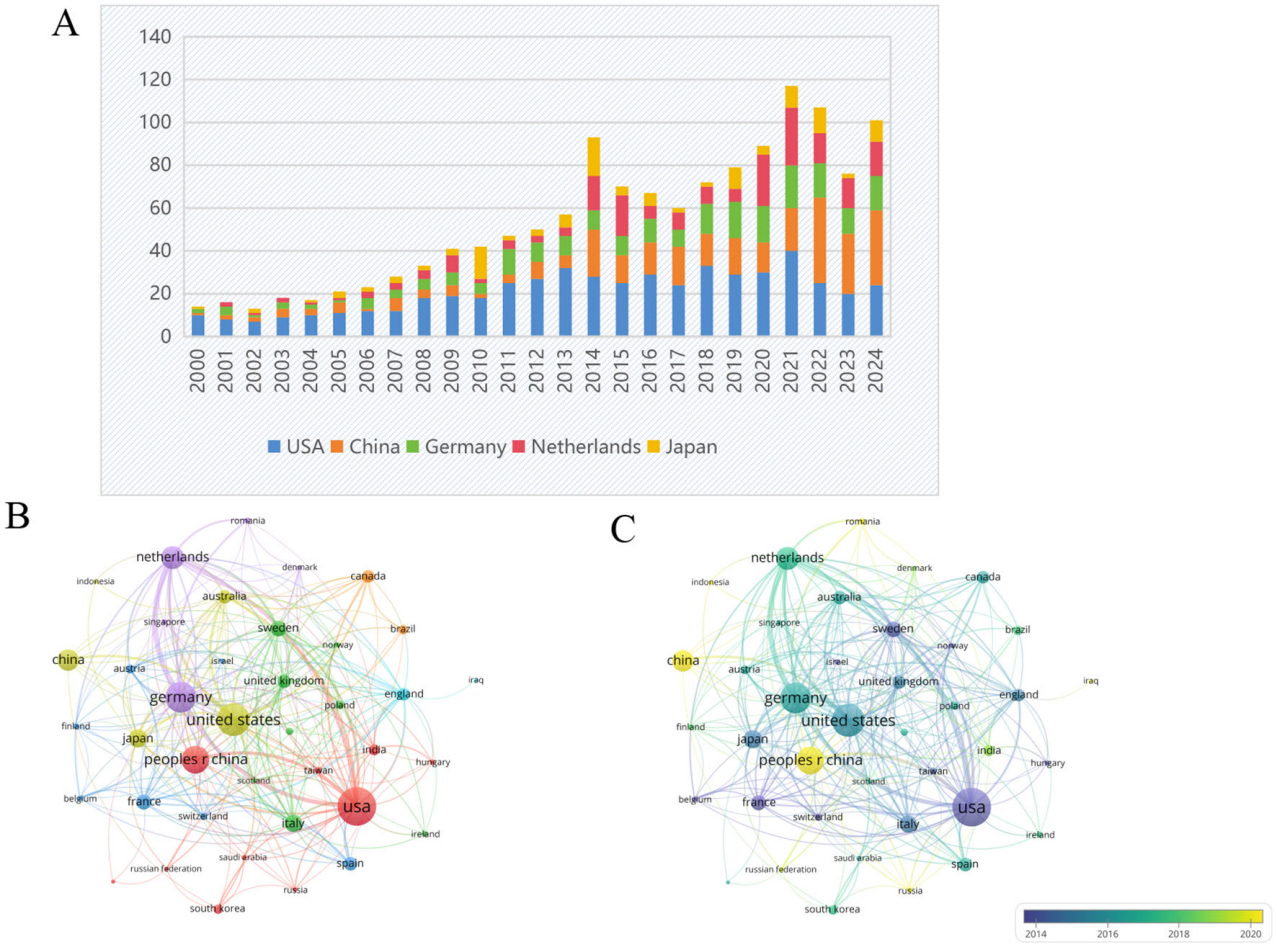
**(A)** Bar graph of the top five productive countries/regions. **(B)** Network visualization map of the top 30 countries’ collaboration. **(C)** Overlay visualization map. From: VOSviewer.

**Figure 7** illustrates the volume of collaborative publications across the 90 countries and regions examined in this field. **Figure 8A** presents a comparative analysis of the number of publications from the top five countries and regions. **Figure 8B** depicts the network diagram of cooperative publications among countries with more than five collaborative contributions in this area. Furthermore, **Figure 8C** indicates that China has engaged in a greater extent of cooperation within this field during recent stages.

### 3.6 Analysis of a highly cited study

**Figure 9:**
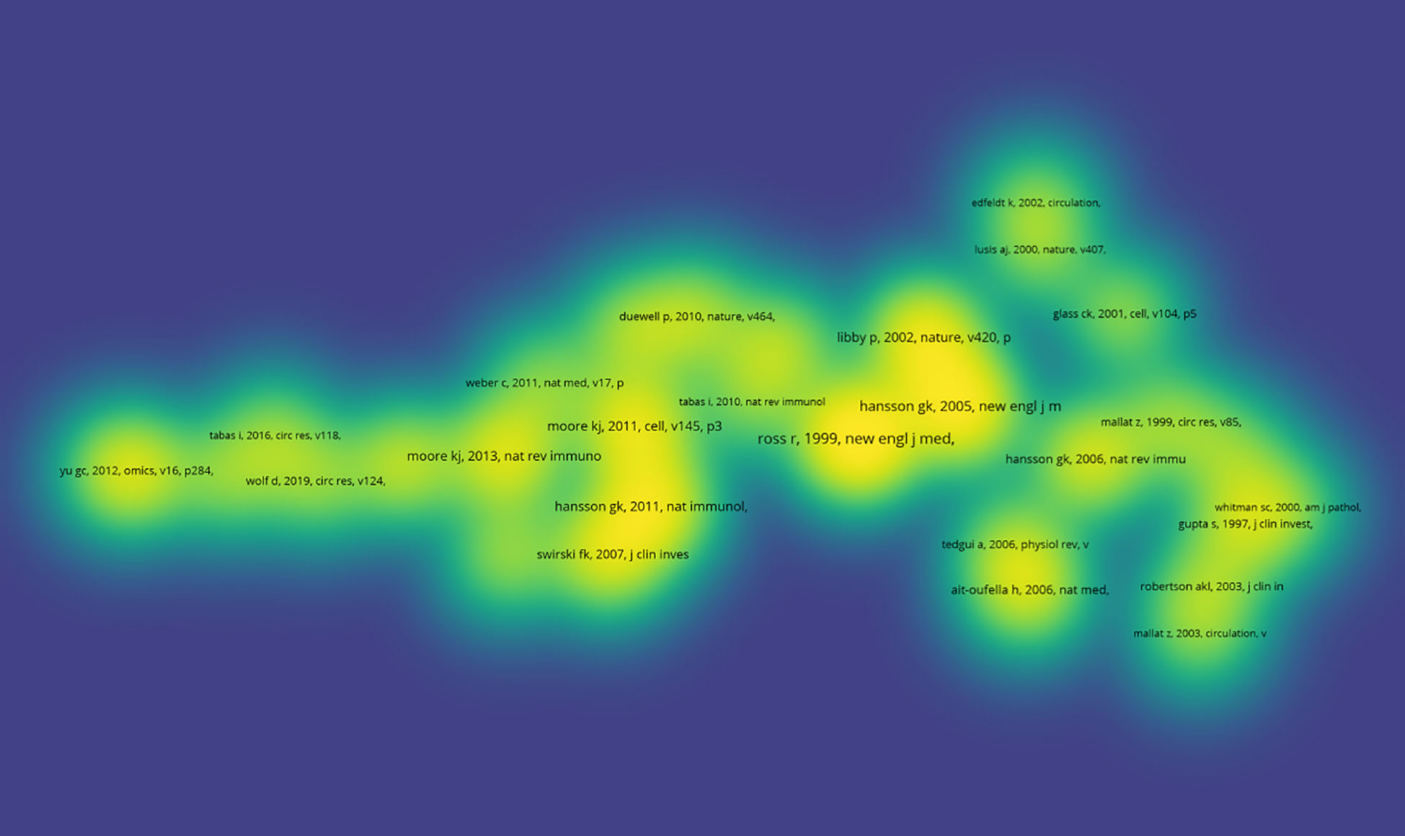
Density visualization map of the co-cited references. From: VOSviewer.

**Table 2** presents the ten most frequently cited articles in research within this field. The article titled “Transforming growth factor-β regulation of immune responses” (19) published in the Annual Review of Immunology has received the highest number of citations. Additionally, **Figure 9** illustrates the co-citation density map for articles that have been cited more than 20 times.

**Table 2:** The top 10 most cited articles.

Rank	Year	First author	Title	Source	Citation
1	2006	Li, Ming	Transforming growth factor-β regulation of immune responses	Annual Review of Immunology	1936
2	2014	Palazon, Asis	HIF Transcription Factors, Inflammation, and Immunity	Immunity	935
3	2016	Feng, Shaolong	The health effects of ambient PM2.5 and potential mechanisms	Ecotoxicology and Environmental Safety	866
4	2006	Zelcer,Noam	Liver X receptors as integrators of metabolic and inflammatory signaling	Journal of Clinical Investigation	836
5	2006	Karin, Michael	Innate immunity gone awry: Linking microbial infections to chronic inflammation and cancer	Cell	834
6	2022	Fahed, Gracia	Metabolic Syndrome: Updates on Pathophysiology and Management in 2021	International Journal of Molecular Sciences	758
7	2008	Bensinger, Steven J	Integration of metabolism and inflammation by lipid-activated nuclear receptors	Nature	713
8	2001	Xu, Xiaoou	Toll-like receptor-4 is expressed by macrophages in murine and human lipid-rich atherosclerotic plaques and upregulated by oxidized LDL	Circulation	606
9	2022	Kong, Peng	Inflammation and atherosclerosis: signaling pathways and therapeutic intervention	Signal Transduction and Targeted Therapy	554

### 3.7 Analysis of keywords

**Figure 10:**
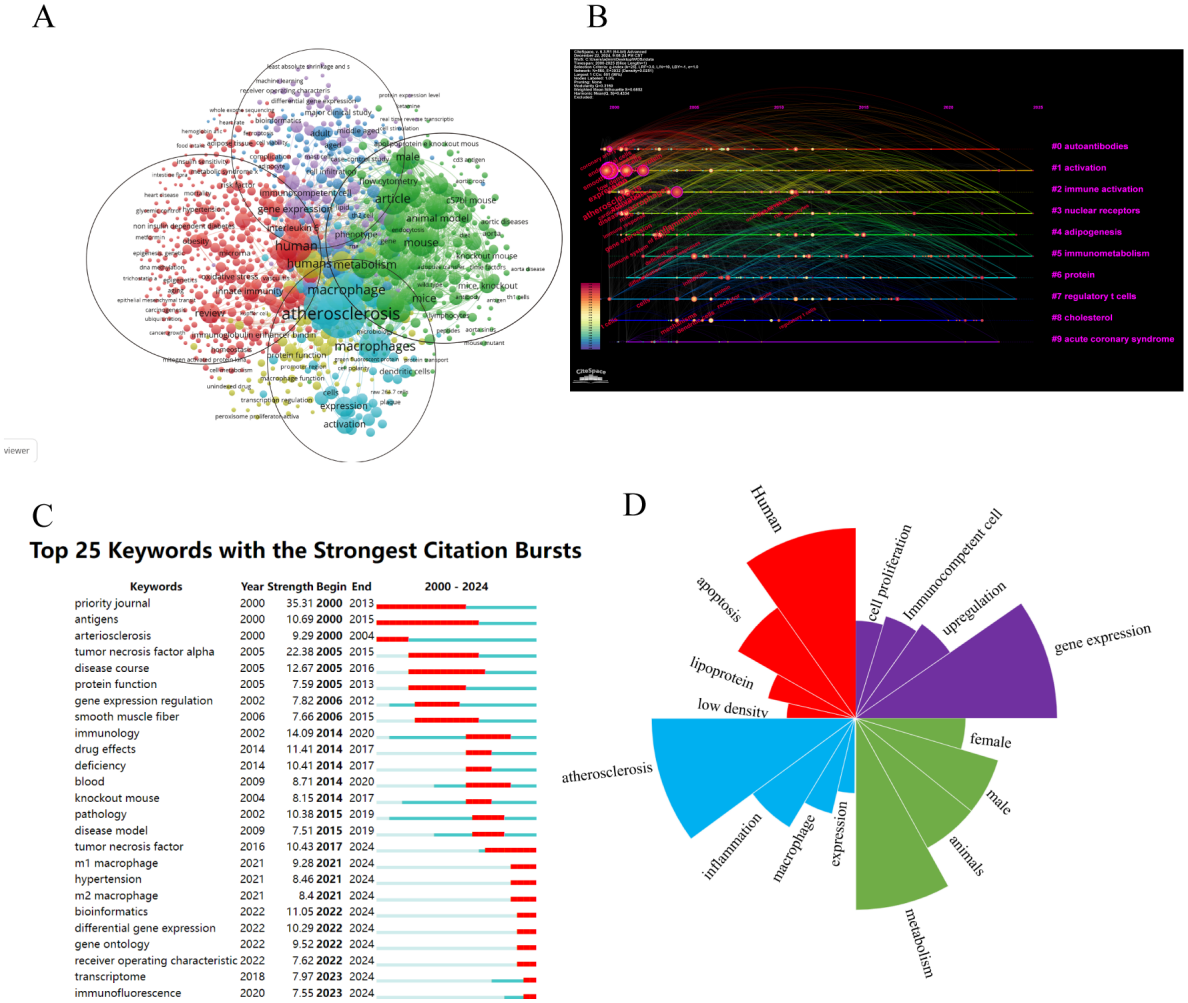
**(A)** A network visualization map. **(B)** A timeline and keyword clustering display. **(C)** Key word mutation top 25. **(D)** Keywords that are particularly important in the four groups.

**Figure 10A** presents keywords with a frequency exceeding 10. From a total of 1469 studies, we identified 136 high-frequency keywords, which were categorized into five distinct clusters. For our analysis, we selected four major clusters out of the five identified. Red cluster: Autoantibodies; keywords include human, apoptosis, low-density lipoprotein, and oxidized LDL. Blue cluster: Activation; keywords are AS, inflammation, macrophage, expression, and activation. Green cluster: Immune activation; this cluster features keywords related to metabolism, animals, male and female subjects, controlled studies, and mice. Purple cluster: Nuclear receptors; relevant keywords are gene expression, upregulation, immunocompetent cells, and cell proliferation. The time chart illustrates the temporal trends associated with various research keywords over time as depicted in **Figure 10B**. Recent research hotspots include gene ontology, receiver operating characteristic, transcriptome and immunofluorescence. Keyword mutation analysis can elucidate the emerging research hotspots within a specific field during designated periods. This is demonstrated in **Figure 10C**, which highlights the top 25 keywords exhibiting the most significant citation surges. In **Figure 10D**, we present the top five keywords with the highest occurrences across the four primary clusters.

## 4 Discussion

### 4.1 Global research status and trends

**Figure 11:**
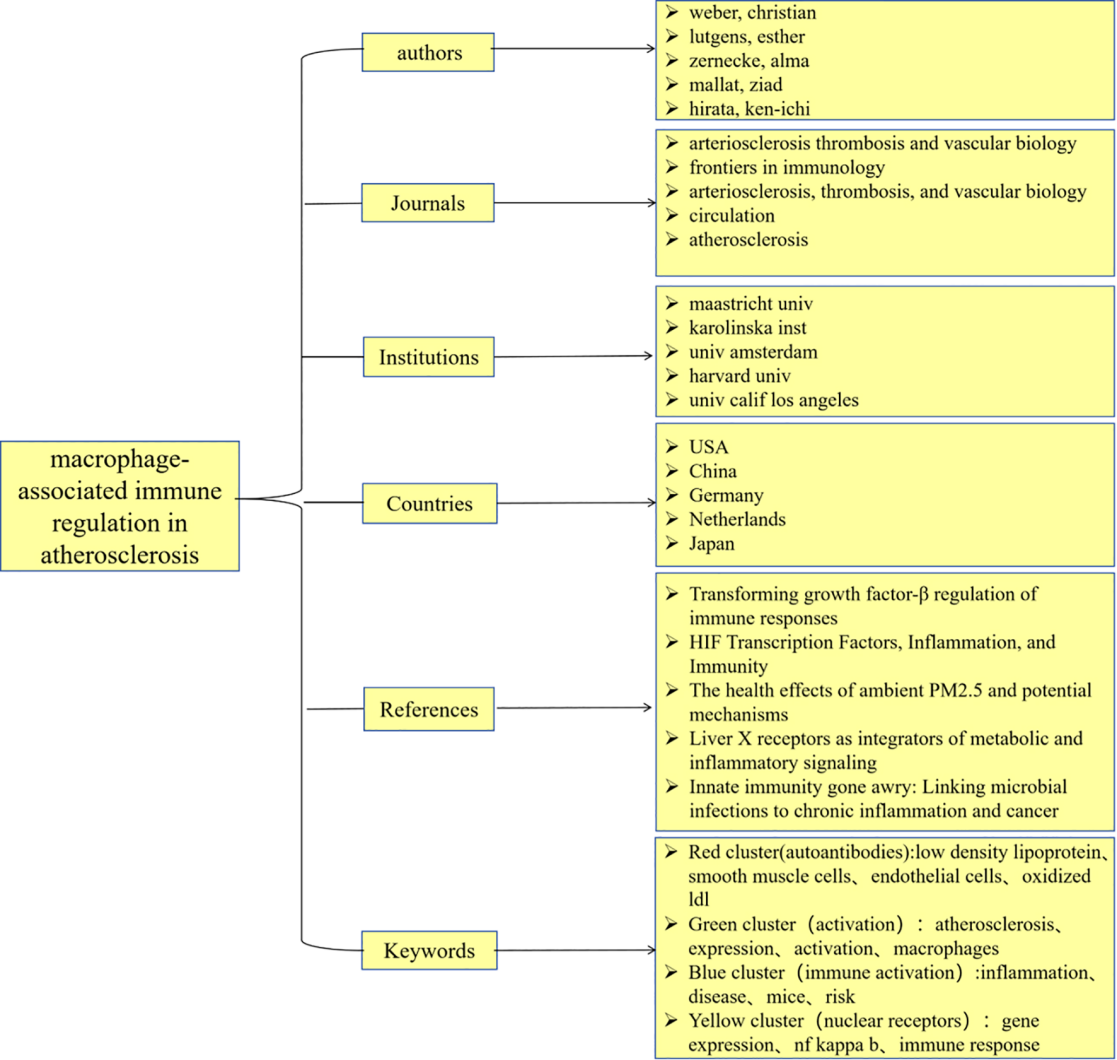
Conceptual framework map.

From the perspective of national publications, the United States, China, and Germany collectively accounted for a total of 598 publications, representing 70.25% of the overall publication output, which highlights the strong scientific research capabilities and significant financial investments in the biomedical sector of these countries. In terms of authorship, all three leading authors are from Germany, highlighting the formation of a close-knit and influential research community in this field within that country. Regarding journal publications and citations, “Arteriosclerosis Thrombosis and Vascular Biology” stands out as a significant journal in this domain, possessing considerable importance and reference value. An analysis of the top ten institutions by publication volume reveals that these institutions are predominantly located in developed countries across Europe and the United States. The leading three institutions identified are Maastricht University, Karolinska Institute, and Universiteit van Amsterdam. The above results are shown in the **Figure 11**.

### 4.2 Research keyword analysis

Keywords are fundamental to a research paper, as they effectively convey the central topic (6). Through cluster analysis of 136 keywords that appeared more than ten times, we categorized them into four distinct groups, each represented by a different color: Red cluster): autoantibodies; Blue cluster): activation; Green cluster): immune activation; and Purple cluster): nuclear receptors. The co-occurrence analysis of these keywords illuminated the primary research directions and trending topics among researchers in this field. By examining the temporal distribution of keywords alongside the citation surge trends for the top 25 keywords, we identified that recent studies have concentrated on inflammation, immune infiltration, mechanisms, macrophage polarization, and dysfunction. Research in emerging areas offers new directions for managing AS. Additionally, developing drugs that target immune activation pathways could yield fresh insights into treatment strategies. Through an in-depth analysis of these four clusters, we explored the research hotspots and emerging trends within the domains of AS and macrophage immune regulation while providing strategic guidance for future investigations.

#### 4.2.1 Red cluster: autoantibodies

After the onset of AS, the involvement of the adaptive immune system significantly accelerates pathological progression. AS induces a breakdown in tolerance to its own components, which is manifested by defective expression of immune checkpoint molecules, dysfunction in antigen presentation, and increased production of autoantibodies (20). Severe congenital and adaptive immune disorders—characterized by type I interferon overload; abnormalities in macrophage function; activation of platelets and complement; dysregulation of neutrophils; formation of neutrophil extracellular traps; uncontrolled T cell activation; as well as excessive production of autoantibodies and formation of immune complexes—have been proposed to promote accelerated CVD in systemic lupus erythematosus (SLE) patients (21). Positive rheumatoid factor (RF) and antinuclear antibody (ANA) serve as predictors for cardiovascular events and mortality among individuals both with rheumatic diseases as well as those without. These findings underscore the role that immune dysregulation plays in the etiology of CVD. In rheumatoid arthritis (RA) patients who possess autoantibodies, there is an acceleration in AS compared to RA patients lacking these antibodies. Similarly, the presence of autoantibodies within SLE also correlates with an increase in atherosclerotic development (22).The role of vascular endothelial growth factor (VEGF) in accelerated AS in SLE Patients (23).; Gene stimulator (STING) is a cytosolic DNA sensor that plays a significant pathogenic role in various inflammatory diseases. Liu Y et al. emphasized the pathogenic involvement of STING and its downstream interferon responses in TLR7-driven autoimmunity, vascular injury, and AS (24). The sequestration of oxidized modified low-density lipoprotein (ox-LDL) by macrophages results in the accumulation of fatty deposits within the arterial wall. The necrosis of these cells leads to the release of intercellular epitopes and activates the adaptive immune system, which Joshua et al. predicted would culminate in robust autoantibody production (25). The autoimmune nature of AS is further substantiated by the development of antibodies against ox-LDL, as well as through experimental induction models involving T cell transfer or immunization with autoantigens such as β2 glycoprotein I (β2-GPI) and heat shock proteins (HSP) (4).Rheumatic autoimmune diseases increase the risk of accelerated AS and various vascular lesions (26).

#### 4.2.2 Blue cluster: activation

Inflammation and immune dysfunction, characterized by the infiltration of classically activated macrophages (M1), are critical mechanisms underlying the progression of AS (27). In the context of AS, lysosomal dysfunction leads to alterations in macrophage cytokine secretion, partially mediated through inflammasome activation (28). Furthermore, N6-methyladenosine (m6A) modification influences macrophage function by affecting their development, activation and polarization processes. It also impacts pyroptosis and metabolic disorders within these cells, ultimately contributing to metabolic diseases associated with impaired macrophage functionality—including AS (29). Zhang et al. reported that desmosterol, the most prevalent intermediate in cholesterol biosynthesis found in human coronary artery lesions, plays a pivotal role in atherogenesis and serves as a key molecule that integrates cholesterol homeostasis with immune responses in macrophages (30). Tan et al. demonstrated that monocyte-macrophage dynamics are predominant in the innate immune response. Two plak-specific monocyte subsets exhibit diametrically opposing functions: EREG+ monocytes promote cerebrovascular events, while C3+ monocytes exert anti-inflammatory effects. Similarly, IGF1+ and HS3ST2+ macrophages, characterized by features of classic pro-inflammatory M1 macrophages, were identified and linked to cerebrovascular events (31). TNF-α inhibitors reduce serum levels of TNF-α, C-reactive protein (CRP), interleukin-6 (IL-6), and other inflammatory markers. They also enhance endothelial nitric oxide synthase expression and improve vasodilatory responses to bradykinin. These effects help slow the progression of endothelial dysfunction and AS (32). Interleukin-17 (IL-17), a key proinflammatory cytokine of the adaptive immune system, is produced by a specific subset of Th17 helper T cells. IL-17 plays a crucial role in the pathogenesis of autoimmune and chronic inflammatory diseases, such as AS (33).

#### 4.2.3 Green cluster: immune activation

Immune activation has become an important part of the immune pathogenesis of AS (34). Mohammad et al. discovered that within arterial plaques, HIV infection induces an inflammatory and immune activation state that triggers the NLRP3/caspase-1 inflammasome, leading to tissue damage and monocyte/macrophage infiltration (35). Takeshi et al. suggested that the uptake and metabolism of fatty acids by macrophages are implicated in numerous critical biological pathways, including macrophage immune activation and regulation, as well as pathological conditions such as obesity and AS (36). Xu et al. reported that DHRS9 plays a role in atherogenesis, with its proatherogenic effects mediated through immune mechanisms. Furthermore, it was confirmed that DHRS9 is localized within macrophages present in atherosclerotic plaques (10). Adiponectin is an abundant hormone facilitating communication between adipose tissue, the immune system, and the cardiovascular system. Ioanna et al. found that adiponectin binds to specific receptors on macrophage cell surfaces—adiponectin receptor 1 (AdipoR1) and AdipoR2—to activate downstream signaling cascades which induce protective functions against atherogenesis (37). Kenneth et al. demonstrated that following immune activation, both macrophages and neutrophils produce angiotensin- converting enzyme (ACE), significantly influencing myeloid cell function independently of angiotensin II levels. Mice genetically modified to express ACE at elevated levels (ACE 10/10) exhibit increased ACE expression in their macrophages; these mice show enhanced resistance to models of AS (38). Tetsu et al. illustrated that niacin inhibits AS by activating the anti-inflammatory G protein-coupled receptor GPR109A expressed on immune cells; this action attenuates both immune activation and adventitial responses (39). Immunometabolism has gained attention in exploring therapeutic pathways for CVD. Metabolic changes in immune cells, triggered by various stimuli, can affect their responses and local signaling. Itaconate, an intermediate of the tricarboxylic acid (TCA) cycle, is involved in cellular metabolism, oxidative stress, and inflammatory responses. The gene immune response gene 1 (IRG1), which encodes the enzyme for itaconate production, is upregulated in activated macrophages. Itaconate and its derivatives exhibit cardioprotective effects through immunomodulation, highlighting their potential as therapeutic agents for CVD (40). Sirtuins (SIRT1-SIRT7), as a NAD+ -dependent protein modifying enzyme family, is a new target for the immune metabolism of macrophages in CVD (41).

#### 4.2.4 Purple cluster: nuclear receptors

Different members of the nuclear receptor (NR) superfamily have been extensively studied as potential therapeutic targets for CVD. The atheroprotective effects of the estrogen receptor (ER) are attributed to its beneficial actions in endothelial cells, smooth muscle cells (SMCs), and macrophages. In addition, vitamin D metabolism and the vitamin D receptor (VDR) are crucial in the cardiovascular system. The enzyme linked to VDR is widely expressed, and VDR activation has shown antiatherogenic effects (42).Nuclear receptors and their cofactors play a crucial role in regulating key pathophysiological processes involved in the development of AS. The transcriptional activity of these nuclear receptors is modulated by nuclear receptor corepressors (NCOR). Oppi S et al. demonstrated that macrophage NCOR1 inhibits PPARγ’s proatherogenic function in AS. This suggests that stabilizing the NCOR1-PPARγ binding may be a promising strategy to hinder both the proatherogenic activities and lesion progression of plaque macrophages in AS patients (43). Phuong et al. reported that activation of nuclear factor-κB and TANK-binding kinase 1 is associated with interferon gene stimulator (STING), a cytosolic DNA sensor linked to vascular inflammation and macrophage activation. STING stimulates proinflammatory activation in macrophages, contributing to the progression of AS (44). As a therapeutic intervention, Prakash et al. reported that inhibiting PKM2 nuclear translocation through small molecules decreased glycolytic rates, enhanced exocytosis, and mitigated AS in Ldlr^−/−^ mice (45). Furthermore, Liu et al. revealed that macrophage calcin-activated T cell nuclear factor (NFAT) c3 (NFATc3) upregulates miR-204 to downregulate SR-A and CD36 levels, thereby preventing foam cell formation and subsequent development of AS (46). Chai H et al. found that zedoarondiol improved AS plaques by modulating the CXCL12/CXCR4 pathway through reduced expression of phosphatidylinositol 3-kinase (PI3K), protein kinase B (AKT), and nuclear factor-kappa B (NFκB) (47). Inflammation and immune dysfunction characterized by infiltration of classically activated macrophages (M1) are significant mechanisms driving the progression of AS (27).

#### 4.2.5 Inter-cluster overlap and interaction

Macrophage-associated immune regulation in AS involves mechanisms such as autoantibodies, inflammatory activation, macrophage activation, immune metabolism, and nuclear receptors. Activation and immune activation are categorized into distinct clusters; however, they are intricately interconnected and play a significant role in the pathophysiological processes of AS. Inflammation and immune dysfunction, marked by M1 phenotype macrophages, are key mechanisms driving AS progression (27). Macrophage accumulation and activation are central to AS. Dysregulation of macrophage polarization between pro-inflammatory M1 and anti-inflammatory M2 phenotypes significantly impacts the immune response during AS progression. The immune metabolism of macrophages is closely linked to their activation and metabolic changes in atherosclerotic lesions, affecting both immune function and tissue repair. Targeting macrophage phenotype and metabolism offers a promising therapeutic strategy for preventing and treating AS (31). Crosstalk between sterol metabolism and inflammatory pathways significantly influences atherosclerosis development. Cholesterol biosynthesis intermediates act as key immune regulators for macrophages during innate immune activation and lipid overload. Alberto Canfran-Duque et al. found that 25-Hydroxycholesterol (25-HC) modulated Toll-like receptor 4 signaling, enhanced nuclear factor-κB-mediated proinflammatory gene expression, and increased apoptosis susceptibility by amplifying the inflammatory response in lipid-laden macrophages (48). Song (49) et al. showed that itaconate requires the antioxidant transcription factor Nrf2 to effectively inhibit oxidative lipid-induced macrophage activation *in vitro*. Additionally, itaconate reduces pro-inflammatory responses in macrophages, leading to smaller atherosclerotic lesions *in vivo*. Autoantibodies and circulating immune complexes in SLE may significantly contribute to AS pathogenesis. This occurs through mechanisms such as endothelial cell damage, induction of pro-inflammatory and pro-adhesive phenotypes in endothelial cells, and alterations in lipoprotein metabolism involved in atherogenesis (50).

In addition, advancements in immunotherapy have gained recognition from scholars. Interferon enhances macrophage inflammatory activity, positioning them at the center of the interferon system and leading immune responses (51).Forteza et al. demonstrated that targeting the PDK/PDH axis with DCA disrupts immune function, inhibits vascular inflammation and atherogenesis, and enhances plaque stability in Apoe −/− mice. These findings suggest a promising therapeutic strategy for AS (52). Immunotherapy advancements are expected to greatly improve personalized patient assessments for AS.

## 5 Limitations

There are several limitations to our bibliometric study. First, the scope of this research was confined to English literature, which may have resulted in the exclusion of significant studies published in other languages. Second, due to the cutoff date for this study, recently published high-quality articles may not adequately reflect their impact because of their brief publication duration and low citation frequency. These temporal sensitivity issues may have affected our ability to capture and assess the most recent research findings. Future research should consider broadening the language range of the literature and periodically updating the database to incorporate emerging research developments.

## 6 Conclusion

Based on bibliometric analyses of macrophage-related immune regulation in AS, we have determined that this field is garnering increasing attention from researchers. In terms of both publication volume and quality, the United States stands out as the most influential country in this domain. A comprehensive examination of publications and citations revealed that “Arteriosclerosis Thrombosis and Vascular Biology” is the leading journal in terms of impact. According to annual publication data, the number of studies focusing on macrophage-related immune regulation in AS has exhibited fluctuations since 2000, peaking in 2024. Consequently, we anticipate that research into macrophage-related immune regulation within the context of AS will remain a focal point for future investigations. Through keyword analysis, we identified four primary research directions within the realm of macrophage-related immune regulation in AS and assessed their corresponding trends. The exploration of immune regulatory mechanisms involving macrophages in relation to AS has emerged as an increasingly prominent area of study. Furthermore, advancement of immunotherapy development has increasingly garnered recognition from a growing number of scholars. Macrophages play a pivotal role in immune regulation throughout the progression of AS. Regulating the immune function of macrophages may represent a novel approach to advancing immunotherapy for AS.

## Data Availability

The original contributions presented in the study are included in the article/supplementary material. Further inquiries can be directed to the corresponding author.
